# Epigenetics of Meningiomas

**DOI:** 10.1155/2015/532451

**Published:** 2015-05-25

**Authors:** Balázs Murnyák, László Bognár, Álmos Klekner, Tibor Hortobágyi

**Affiliations:** ^1^Division of Neuropathology, Institute of Pathology, Faculty of Medicine, University of Debrecen, 98 Nagyerdei Körút, Debrecen 4032, Hungary; ^2^Department of Neurosurgery, Faculty of Medicine, University of Debrecen, 98 Nagyerdei Körút, Debrecen 4032, Hungary

## Abstract

Meningiomas account for one-third of all adult central nervous system tumours and are divided into three WHO grades. In contrast to the relatively well characterized genetic alterations, our current understanding of epigenetic modifications involved in the meningioma-genesis and progression is rather incomplete. Contrary to genetic alterations, epigenetic changes do not alter the primary DNA sequence and their reversible nature serves as an excellent basis for prevention and development of novel personalised tumour therapies. Indeed, growing body of evidence suggests that disturbed epigenetic regulation plays a key role in the pathogenesis of meningiomas. Altered DNA methylation, microRNA expression, histone, and chromatin modifications are frequently noted in meningiomas bearing prognostic and therapeutic relevance. In this review we provide an overview on recently identified epigenetic alterations in meningiomas and discuss their role in tumour initiation, progression, and recurrence.

## 1. Introduction

Meningiomas originate from arachnoidal cap cells of the leptomeninges and account for around 30% of all central nervous system (CNS) tumours in adults. Their incidence increases with age with a female : male ratio of 2 : 1. Furthermore, autopsy and imaging studies indicate that the prevalence of subclinical meningiomas is roughly 3% in the population [1]. According to the World Health Organization (WHO) meningiomas can be divided into three histological grades: benign (grade 1), atypical (grade 2), and anaplastic meningiomas (grade 3). Besides this classification several other subtypes and variants exist (Table 1) [2, 3]. Although, 90% of meningiomas are typically benign slow-growing tumours, grade 2 and 3 tumours exhibit aggressive clinical phenotype with an increased risk of recurrence and invasive growth pattern [4]. The main histopathological characteristics of the different grades and subtypes were thoroughly reviewed by Riemenschneider et al. [5].

**Figure 1:**
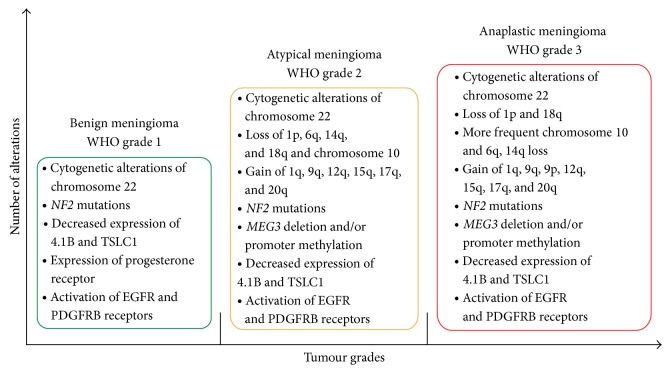
Characteristic cytogenetic and molecular alterations of the different WHO grades of meningiomas.

However, most meningiomas are sporadic; they are rarely associated with familial tumour syndromes such as Li-Fraumeni, Turcot, Gardener, von Hippel-Lindau, Cowden, Gorlin, and multiple endocrine neoplasia type I (MEN1) [6]. The aetiology of meningiomas is still unclear; ionizing radiation, head trauma, hormone-replacement therapy, and advanced age are the established risk factors though [7]. While the cytogenetic landscape of tumours is well known, the molecular mechanisms underlying progression and recurrence are not well defined. Cytogenetic alterations of chromosome 22 and* NF2* gene are characteristic genetic alterations in early tumorigenesis and are frequently noted in higher grade tumours. In contrast to benign meningiomas, grade 2 and 3 tumours display more complex cytogenetic and molecular background with activation of oncogenes, inactivation of tumour suppressor genes, and alterations in other genes involved in several cellular pathways (Figure 1) [8]. Despite the identification of many potential molecular targets current treatment strategies are still limited to conventional forms of tumour therapy [9].

During the last decade, disruption of normal epigenetic regulation has been recognized as a novel hallmark of cancer [10]. Deeper understanding of epigenetic modifications in meningiomas may increase our knowledge regarding tumorigenesis, progression, and recurrence. The goal of our review is to provide an overview of advances in the field of meningioma epigenetics with focus on possible biomarkers which may open the door to novel and more effective molecular diagnosis and therapies.

## 2. Epigenetic Profile of Meningiomas

In the past cancer was viewed as a disease initiated by the accumulation of genetic alterations causing neoplastic transformation in the respective cell type of origin. However, in the new millennium, it has become evident that also epigenetic regulation plays a prominent role in tumorigenesis. In principle, epigenetic modifications are heritable changes that influence gene expression without altering the primary sequence of DNA [11]. Besides cancer, disruption of the normal epigenetic regulation may also contribute to the pathogenesis of inflammatory, autoimmune, metabolic, neurological, and blood disorders [12]. Since the basics of epigenetics in cancer had been established it has become one of the most promising research fields of neurooncology which may reveal potential targets for drug development and therapy [13]. However, the epigenetic landscape of meningiomas remains still incomplete with altered DNA methylation, aberrant microRNA expression, and mutant epigenetic modifiers (EMGs) involved in histone and chromatin modifications being potential epigenetic markers of progression and recurrence (Table 2).

### 2.1. Aberrant DNA Methylation in Meningiomas

**Figure 2:**
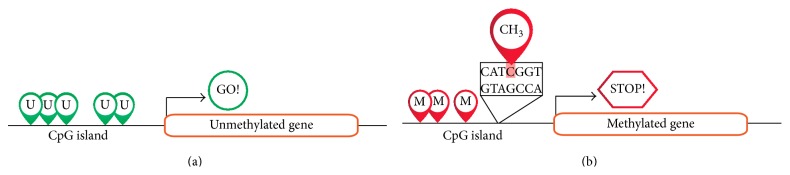
Schematic representation of unmethylated (a) and methylated (b) genes. The most CpG islands in the promoter region of normal genes are unmethylated (a) [11]. DNA methylation refers to the addition of a methyl (CH_3_) group to the fifth carbon atom of the cytosine residues resulting in the formation of 5-methylcytosine (b). The process is mediated by DNA methyltransferase enzymes. DNA methylation occurs mainly at cytosine-guanosine dinucleotides (CpGs) which are concentrated in promoter CpG islands. CpG islands are short DNA sequences (<200 bp) with greater than 50% GC content. Methylation of CpGs in promoter regions plays an important role in both chromatin structure control and gene expression [14].

Altered DNA methylation was the first epigenetic mark shown to be associated with cancer caused by both global DNA hypomethylation and/or promoter hypermethylation of certain genes.* De novo* methylation of DNA is catalysed by the enzymes DNMT3A and DNMT3B whereas maintenance of methylation is mediated by DNMT1 (Figure 2) [14, 15].

Intriguingly,* NF2* (neurofibromatosis-2; a gene known to be frequently involved in development of meningiomas) promoter methylation does not play a key role in meningioma development [16]. Nevertheless, emerging evidence supports the involvement of DNA methylation in meningioma progression [13]. In contrast to* NF2*, the promoter methylation of tissue inhibitor of metalloproteinase 3 (*TIMP3*), located quite close to the* NF2* gene, is inactivated in meningiomas [17]. Hypermethylation of* TIMP3* is present in 67% of anaplastic meningiomas, but only in 22% of atypical and 17% of benign meningiomas. Thus, inactivation of* TIMP3* by methylation may be involved in meningioma progression and can be a potential marker of an aggressive, high-grade meningioma phenotype [18]. Similar to* TIMP3*, repression of* HOXA7*, *HOXA9,* and* HOXA10*  in meningioma is also associated with clinically aggressive behaviour. DNA methylation levels of* HOXA7*, *HOXA9,* and* HOXA10*  were reported to be significantly higher in atypical and anaplastic meningiomas than in the benign form [19]. The methylation status of these three genes was lower in newly diagnosed grade 1 meningiomas in contrast to their recurrent counterpart and multiplex meningiomas presented with significantly higher* HOXA10* methylation as compared to solitary meningiomas [20]. Promoter methylation of* RASSF1A*,* TP73,* and* NDRG2* is more frequent in higher grade tumours than in benign meningioma [21, 22]. Repressed O^6^-methylguanine-DNA methyltransferase (MGMT) by promoter methylation is a prognostic biomarker in glioblastoma (GBM) [23]. The alkylating chemotherapeutic agent temozolomide (TMZ) increases the overall survival in GBM patients where the* MGMT* gene is methylated [24]. Unlike the vast majority of gliomas, testing the* MGMT* methylation status is not essential because the gene is unmethylated in the majority of meningiomas [25]. On the other hand, Larijani et al. found an increased but statistically not significant* MGMT* methylation with higher tumour grade, it was more frequent in males [26].

High-throughput techniques have made the genome-wide methylation analysis of human tumours including meningiomas possible. Evidence suggests that anaplastic meningioma could be distinguished from atypical and benign tumours according to DNA methylation patterns [19]. In addition, unlike in benign meningiomas, grade 2 and 3 tumours demonstrate increased global DNA hypomethylation. Interestingly, the majority of hypermethylated genes are suppressed in all tumours, but* MAL2* is highly expressed in grade 1 and silenced in grade 3 meningiomas [27]. Another study identified nine differentially methylated genes by whole genome methylation analysis of benign and atypical meningiomas. The largest difference in methylation status was observed in* IGF2BP1* and* PDCD1* [28].* IGF2BP1* encodes the IGF2 mRNA binding protein 1 (IGF2BP1) which mediates the cytoplasmic fate of specific target mRNAs including ACTB and CD44. IGF2BP1 is a potent oncogenic factor that regulates the adhesion, migration, and invasiveness of tumour cells by modulating intracellular signalling [29].

Using genome-wide methylation analysis, grade 1 and 2 meningiomas can be divided into three subgroups. Based on this result, a simplified scoring system with five hypermethylated genes (*HOXA6*,* HOXA9*,* PENK*,* UPK3A,* and* IGF2BP1*) was proposed. This classification correlates well with recurrence and progression but there was no association with WHO histological or Simpson neurosurgical grades [30].

### 2.2. Mutations Related to Histone Modifications

Histone modifications are disturbed in many diseases including cancer. Histone octamer cores are formed by two copies of each histone protein (H2A, H2B, H3, and H4) and one H1 linker histone [31, 32]. Besides DNA condensation, histone proteins are also involved in regulation of gene expression by posttranslational modifications which mainly occur along their N-terminal tail. Histones can be modified by acetylation, methylation, phosphorylation, sumoylation, and ubiquitination. These modifications can result in an open (euchromatin) or closed (heterochromatin) state of the chromatin [31, 33]. Hence, methylated H3K9, H3K27, and H4K20 can result in a closed chromatin conformation, while open chromatin structure can be caused by methylated H3K4, H3K36, and H3K79 [34].

Not much is known about the precise role of these modifications in the initiation and progression of cancer. Histone modification pattern such as histone H3 lysine 9 trimethylation (H3K9me3), which has a prognostic relevance in glioblastoma, has not been detected in meningiomas yet [35]. On the other hand, current genome-wide studies reported mutations of epigenetic modifier genes encoding proteins that regulate the chromatin structure of cells. Among the affected EMGs* KDM5C* and* KDM6A* are histone demethylases, while* SMARCB1* and* SMARCE1* are members of the SWI/SNF chromatin-remodelling complex [36, 37].

### 2.3. Role of MicroRNAs in Meningiomas

MicroRNAs (miRNAs) are single-stranded noncoding RNAs, composed of 19 to 24 nucleotides, and play regulatory roles in important biological processes, such as cell cycle, proliferation, differentiation, migration, and apoptosis [38]. They cause posttranslational gene expression silencing by binding to complementary sites on their target mRNAs and initiate either degradation or inhibition of translation. Dysregulated miRNAs expression was described in a number of human diseases, including cardiovascular, autoimmune, inflammatory, neurodevelopmental diseases and cancer [39, 40]. Altered miRNA expression is associated with both genetic and epigenetic mechanisms. miRNAs usually have reduced levels in tumour cells and probably function as oncogenes or as tumour suppressors [41]. In cancer, several miRNAs correlate well with clinicopathological features, such as metastasis, recurrence, and length of survival [42].

MicroRNA expression profiling of meningiomas revealed downregulation of miR-29c-3p and miR-219-5p, which were associated with advanced clinical stages. High expression of miR-190a and low expression of miR-29c-3p and miR-219-5p are correlated with significantly higher recurrence rates in meningioma patients. Importantly, miR-190a expression level is a prognostic predictor of postsurgical outcomes [43]. The mRNA of *β*-catenin is a target for miR-200a; consequently *β*-catenin translation and Wnt signalling are suppressed by overexpressed miR-200a [44], and miR-200a may act as a multifunctional tumour suppressor miRNA. A direct correlation was found between the downregulation of miR-200a and the upregulation of *β*-catenin in meningiomas. Reduced miR-200a causes decreased expression of the ZEB1 and SIP1 transcription factors resulting in the downregulation of E-cadherin [45]. Moreover, it provokes an increased *β*-catenin and cyclin D1 expression and activates the Wnt signalling pathway in meningiomas [46]. miR-145 may have an important antimigratory and antiproliferative function in cancer [47]. Contrary to grade 1 meningiomas, significantly decreased miR-145 levels were detected in grade 2 and 3 tumours. Increased levels of miR-145 may result in downregulated collagen type V alpha (COL5A1) expression. Accordingly, COL5A1 expression is upregulated in atypical and anaplastic meningiomas [48].

## 3. Conclusion

In the past decade it has become evident that epigenetic factors are involved in tumour pathogenesis. Contrary to genetic alterations, epigenetic changes do not alter the primary DNA sequence and their reversible nature serves as an excellent basis for prevention and development of novel and personalised cancer therapies. Similar to other tumours, disturbed epigenetic regulation plays a key role in the pathogenesis of meningiomas. DNA methylation, microRNA expression, histone, and chromatin modifications can be altered in meningiomas with a prognostic relevance and may become a potential therapeutic target in the future.

## Figures and Tables

**Table 1:** The histological subtypes of meningiomas.

	Histological subtypes
Benign meningioma (WHO grade 1)	Meningothelial, fibrous or fibroblastic, transitional or mixed, psammomatous, angiomatous, microcystic, secretory, lymphoplasmacyte-rich, and metaplastic meningioma

Atypical meningioma (WHO grade 2)	Chordoid, clear cell, atypical, and brain invasive meningioma

Anaplastic meningioma (WHO grade 3)	Papillary, rhabdoid, and anaplastic or malignant meningioma

**Table 2:** Epigenetic changes and their supposed role in meningiomas.

Epigenetic alterations	Affected genes	Distribution between WHO grades	Possible effects in meningioma
Promoter methylation	*TIMP3 *	Grades 1 < 2 < 3	Associated with tumour progression and aggressiveness parameters
	*HOXA7*, *HOXA9, * and *HOXA10 *	Grades 1 < 2 < 3	Associated with tumour progression and aggressiveness parameters
	*RASSF1A *	Grades 1 < 2 < 3	Associated with the malignant transformation of a meningioma
	*TP73 *	Grades 1 < 2 < 3	Associated with the malignant transformation of a meningioma
	*NDRG2 *	Grades 1 < 2 < 3	Associated with malignant progression and predisposition to recurrence
	*MAL2 *	Grade 1	Unknown
	*IGF2BP1 *	Grade 2	Increases the malignant potential of tumours
	*PDCD1 *	Grade 2	Increases the malignant potential of tumours

Disturbed chromatin regulation	*KDM5C *	Grades 1 and 3	Disturbed chromatin regulation
	*KDM6A *	Grade 2	Disturbed chromatin regulation
	*SMARCB1 *	Grade 1	Abnormal chromatin remodelling

Abnormal microRNAs expression	miR-29c-3p	Grades 1 > 2 > 3	Associated with advanced clinical stages
	miR-219-5p	Grades 1 > 2 > 3	Associated with advanced clinical stages
	miR-190a	Grades 1 < 2 < 3	Associated with advanced clinical stages
	miR-200a	Unknown	Functions as a multifunctional tumour suppressor miRNA
	miR-145	Grades 1 > 2; 3	Has an important antimigratory and antiproliferative function
